# Round the Hospitals

**Published:** 1917-08-25

**Authors:** 


### ROUND THE HOSPITALS.

One of the most important items amongst the factors on which the welfare and strength of the British nursing profession in the future depends is undoubtedly the strong position the Nightingale Fund occupies to-day. The wisdom of the Fund's administrators, the administrative and technical knowledge and prowess of Miss Lloyd Still, the appointment of a Sister Tutor who is a graduate of Aberdeen University, the generosity of Mrs. Minet, who endowed a Gold, Silver, and Bronze Medal for competition amongst third-year nurses, which "will open to the holders a wide future of work and interest, apart from the impetus which the medals have given to both pupils and teachers during the courses of study," the momentous developments in the teaching and training of nurses which have resulted, and the energetic cultivation of the opportunities enjoyed by those directly responsible for the present policy of the Fund, all these are noteworthy features. From the opening of the School in June 1860 to the end of last year 2,845 candidates have been admitted; and 1,697 have, after completing a year's training, been entered on the Register of Nightingale Nurses and taken on the hospital staff. During 1916 forty-eight certificates were granted, twenty-three to special probationers (three years), and twenty-five to ordinary probationers. Of the forty-eight certificated nurses twenty-eight are working in the Military Nursing Services, and the remainder in private military hospitals. Four nurses were retained for charge work and work in the maternity ward. The matron is impressed by the return of old Nightingale sisters and nurses for military work, and properly expresses gratitude to the Civil sisters who have so willingly carried on the work of the Civil wards and the teaching of the probationers. The work of the Civil sisters, who have been supported only by very junior staff nurses, under conditions of added strain from the strenuous character of the work, has been necessarily increased. Their successful and self-denying efforts confirm once more the strong claims of the home workers in our hospitals in Great Britain, to remembrance and recognition after the war. Most satisfactory reports of the work done by the many past Nightingale nurses, who are working in the Naval and Military Services and other departments of war work, have been received by the matron. The medical and surgical staff have once again shown great kindness and consideration during the illness of the nursing staff. The examinations for the Gold and other medals, though not mentioned in the report, took place recently. The results will no doubt be intimated as usual when the medals are presented in the autumn. It is satisfactory to find that the Fund has had an excess of income over expenditure for the year. The address of the office of the Royal National Pension Fund for Nurses is wrongly given. It should be Pensions Building, 15 Buckingham Street, Strand, London, W.C.

Our attention has been called to a point which demands the urgent and full care of every matron attached to a hospital where nurses are trained. Twelve hours' continuous work out of each twenty-four as a night nurse, in a great hospital, is a very arduous, and not infrequently a dangerously wearing occupation, for the healthiest of women. Where, as in one case within our experience, an unfortunate probationer was kept on night duty for nine months, and put every night to the task of cutting bread-and-butter, it is as remarkable as it is satisfactory to learn that no evil results to health followed, though the injustice to the nurse who had entered for three years' training, not fooling, must be manifest. We hope the days are past, when any matron will ever again penalise a night nurse because she may be unpopular, or for other personal reasons. There is, however, in some of the larger hospitals a want of consideration and justice where night nurses are concerned, that would amount to a crime, if it did not point to surpassing incapacity on the part of someone in authority. We had the case of one of the best and most deserving of trained nurses brought to our notice quite recently. She was called upon to undergo her *vivâ voce* examination for her final for the certificate on coming off night duty, where she had been for twelve continuous hours. How far similar cases may occur, or may be liable to occur, in the voluntary or other hospitals of this country, we are not in a position to state definitely. It is enough that such cases should ever happen in a British hospital, and we rely upon the matrons throughout the country immediately to inquire into this matter, with a view to the abolition of any existing abuses of the kind. We shall be glad to have the experience and views of any matron or sister who will be kind enough to send them to the Editor, at her convenience.

---

Since May a correspondence on the Registration of Nurses has appeared week by week in the *Sunday Times*. It was started by Miss Mary Macleod Moore, Chairman of the Council of the Canadian Society of Women Journalists. It was supported by the few women associated with the promoters of the Central Committee, the Misses Eden, Paterson, Cartwright, Henrietta Hawkins, Margaret Breay, Beatrice Kent, Mrs. Bedford Fenwick, and "A Lifelong Lover of Justice," and answered by the Misses A. Millicent Ashdown, Vera Matheson, M. A. Bompas, and "A Lifelong Friend of Nurses." Any nurse who is interested may see the correspondence at any time if she calls at our office, 28 and 29 Southampton Street, Strand. This correspondence has resulted in the disposal of every point raised by the Central Committee's supporters, with the exception of a ridiculous charge, made by "A Lifelong Lover of Justice," that Mr. Stanley committed a deliberate breach of faith and honour by deleting a clause inserted in the first Bill drafted by the College of Nursing, Limited. In precise language, the Central Committee insisted on the direct representation of nurses' societies on the First or Provisional Council to be set up under any Bill for the State Registration of Trained Nurses. In the course of discussions on the Bill it was found advisable that the names of those nominated by the various bodies should appear in the Bill, not the names of certain or any societies. The reason for this decision was twofold: (1) It appeared advisable, and in this we are confident our readers will agree, that the nurses should know the names of those who were going to govern them during the first two years under the Bill; and (2) it looked probable that it would be easier to get the Bill through Parliament if it had the names of people instead of societies authorised to nominate. If one society was mentioned as having the right to appoint representatives on the Council, it was certain that half a dozen other societies would claim an equal right. Nurses will understand that the Central Committee under the draft Bill have the right to nominate an equal number of named representatives to that granted to the College. Further, the Central Committee knows that the College Council is quite willing at any time to resume negotiations for an agreed Bill, if they ask the College to do so. The "deliberate breach of faith and honour" so becomes demonstrably a myth. No vital principle is involved in the direct representation of nurses' societies, but **the vital principle** for which the College and the great body of nurses are contending, **is the direct representation of the nurses themselves.** Nurses who read the correspondence in the *Sunday Times* will find that it contains facts concerning some of the societies represented on the Central Committee, which prove them to have no real claim to representation at all. We hope, therefore, that British nurses, or the overwhelming majority of them, will agree, that the vital principle claimed to vest in representation through societies, does not and cannot exist in fact.

On p. 420 will be found a thoughtful article by the writer of "Sparks from a Nurse's Anvil," which we hope may be widely read and pondered. We should like to have some correspondence on the points there raised as to the standard and actions of individual nurses in the course of their work. Especially and properly does our correspondent condemn the want of individual standards, and the absence of courage to do what is right and best. Do not shiver on the bank or try always to please, what she denominates, the Management. The maintenance of standards does not mean aggressive conduct, but merely a firm adherence to principle, not only in our relations with each other within the hospital walls, but in regard to the maintenance of a high standard by all and sundry, over whom we may have some influence or control, or both. The very able matron who contributes the article to "Matrons in Council" this week rightly attaches the first importance, in nursing matters, to the love and sympathy of a noble woman. Every woman attracted to nursing, as a career, must surely possess sympathy, and its practice tends to nobility both of character and conduct.

Meanwhile our nurses are learning with commendable zeal many new lessons, and out of the knowledge which is coming to them will undoubtedly arise a spirit of resistance to systems, which overtax the individual nurse, her strength, her life, and her efficiency. Bad as excessive hours may prove to a nurse working in an institution, their worst feature and frequent danger is that they tend, as our correspondent properly insists, to the formation of bad habits which, when indulged in in private work may prove fatal to a nurse's successful career, and even worse things. Hospital authorities in this country are beginning to awaken to the urgent need of a reconstruction of hours of work for nurses and members of the resident staff. With the College launched and prospering, it is certain that existing anomalies and wrong conditions will be speedily adjusted or abolished altogether.

We incline, however, to the opinion that the private nurse, whose average earnings should not be less than from £90 to £120 a year, if she is really competent and capable, should be in a position to make provision against a rainy day, and to maintain a permanent address to which she can return after work or when in need of rest, with the assured certainty that she will find there every reasonable comfort and a home for herself. Our correspondent appeals to the matrons, and we know she will not appeal in vain. Every week our letter-box contains encouraging evidence of this momentous fact in the coming developments in the British nursing profession and its uplifting. On the other hand, is not our correspondent a little too much absorbed in the feelings and wants of the nurse, to the exclusion of all other workers in the nursing field? She may not be, but as she is an able writer we suggest the question for her consideration, that what she writes and whenever she writes she may do so from the point of view of an enlarged perspective that embraces the whole field, though she may treat that field in sections or compartments at her will and pleasure.

British nurses, not only in Great Britain, but abroad and throughout the Empire, view with gratification the important step taken by Mr. Arthur Stanley, C.B., M.P., Chairman of the Council of the College of Nursing, Limited, in undertaking to use the utmost efforts of which the College is capable to secure fair and even generous treatment for the nurses, who are doing so much for their country and for the sufferers in this great and terrible war. *The Nursing Mirror* did good service in calling attention to the demands of justice that all military nurses, however employed in war work through the Government and Red Cross organisations, should receive identical pay, allowances, and all other things which would secure to them full justice and acknowledgment. It was

**ROUND THE HOSPITALS—(*continued*).**

time that such a guarantee should be forthcoming that everything possible would be done to promote the health and comfort, and to secure the best services of all British nurses who have the privilege of attending to our warriors and those of our Allies, not only at home and in France, but elsewhere throughout the world.

---

A Sister working in a provincial hospital, after testifying to the importance of The Hospital "owing to its most valuable concise information, and also to the value of the articles on the College and Registration Bills, which have made the position clear to all its readers," suggests certain matters which the College can effectually remedy. She is most anxious that the College should lay down one status of training for all nurses. She wants uniformity in the theory and practice of nursing, and in the conditions, salaries, and uniform of all British nurses. She looks to the College to demonstrate the vital importance to the future nurse, which attaches to the refined and careful instruction and training of all probationers, and makes a great point of enforcing reforms and uniformity of practice for ward sisters, who are charged with the duty of instructing probationers in their wards. She maintains that the ward sister does not invariably devote sufficient time to this training, for want of sufficient leisure to carry out these duties with efficiency. She suggests as a remedy, that every sister whose duties involve the training of probationers shall be selected because of her teaching qualities, given ample facilities as an instructress, and sufficient time to enable her, as a capable trainer, to do this work efficiently. Further, that she shall be given a distinctly higher position than those sisters who are not capable of undertaking training. Our correspondent displays a proper interest in the welfare and success of British nurses, and her suggestions are entitled to have that consideration by the College which we have no doubt they will in due course receive.

---

Mr. Geoffrey Young, officer of the British Ambulance Unit for Italy, and formerly in command of the French Unit at Ypres, has written from Gorizia to the *Times* the following interesting account of Sister Julienne, whose lamented death by a shell has been universally regretted: "Sister Julienne, one of the senior sisters of the Civil Hospital at Ypres, has just been reported killed by a shell. This saintly and devoted woman, well on in years, was one of the few heroic sisters who remained in Ypres during the first terrible bombardment, when the town was abandoned. She remained to care for a number of wounded Germans, nursing them until they were finally removed. A few days later she was one of those who returned when the Friends' Ambulance Unit opened in the cellars a ward for the wounded children and civilians. She remained through all the subsequent bombardments; one of the boldest fighters of the epidemic, one of the most courageous to issue under shell fire to fetch in the wounded and the sick. When the town was finally abandoned, she remained with one other sister, on her own initiative, at an aid-post in the cellars, to nurse the wounded British soldiers. Only under compulsion did she at last retire to Poperinghe, where she remained to the end, nursing civilians and soldiers alike, under repeated bombardments, until her death. She was the custodian of the treasures of her hospital, and braved death constantly to visit or remove them. A gentle, sensitive, nervous lady, she was beloved by the wounded children for her sunny gentleness, and by those who worked with her for her refinement and humour. Nothing but a profound sense of duty and love could have supported her through peril and shock, sight and sound, such as proved too much for vigorous men. There may be many heroines in the war more conspicuous, but none whose courage was more tested, or whose motives and character were more beautiful." Sister Julienne was indeed a heroine whose life will inspire and hearten her sisters everywhere throughout the world. Her tomb may with absolute justice bear the epitaph: "She did what she could, *in arduis fidelis*."

---

Miss M. R. Trueman, a new lady superintendent of Queen Alexandra's Military Nursing Service for India, first worked in that country during the plague of 1897, when her work was excellent and highly commended. In the following year, 1898, she joined the Indian Military Nursing Service, and will complete her twenty years' continuous service if she lives for another year. Miss Trueman has well earned her promotion to the high and important post of a lady superintendent of Queen Alexandra's Military Nursing Service for India. Her appointment cannot fail to give pleasure and satisfaction to the authorities of the Hitchin Infirmary and the Royal United Hospital, Bath, who both share the honour of having had a hand in her training and certification as a British nurse.

---

☛ It is our invariable practice to pay for all items of news which we may publish. The minimum payment is 5s. Paragraphs of about twenty lines in length—that is to say, of 150 words or less—are preferred. In this section during the war we propose to give 10s. to the correspondent who may send us in any week the best incident connected with hospital life and work. In this way we hope to afford to all practical aid and encouragement; while those who reciprocate will be able to render us invaluable service. Contributions should be addressed to The Editor, The Hospital, 28 and 29 Southampton Street, Strand, London, W.C., and, to be in time for the current week's issue, should reach him by the first post on Mondays.

**For a Rejoinder to "An Old Shoe's" Thoughts, see p. 420.**
**For Matrons' and Sisters' Appointments, see p. 428.**

